# Higher incidence of aseptic loosening caused by a lower canal filling ratio with a modified modular stem in total hip arthroplasty

**DOI:** 10.1186/s13018-020-02101-x

**Published:** 2020-11-30

**Authors:** Kyosuke Kobayashi, Kenichi Kidera, Masaru Itose, Tetsuhiko Motokawa, Ko Chiba, Makoto Osaki

**Affiliations:** Nagasaki University Hospital, 1-7-1 Sakamoto, Nagasaki, Nagasaki 852-8501 Japan

**Keywords:** THA, Modular prosthesis, Aseptic loosening, Canal filling ratio

## Abstract

**Purpose:**

Although a cementless modular prosthesis has shown reliable results, cases of unstable fixation and revision due to aseptic loosening were observed in our institute. The purpose of this study was to clarify the causes of unstable fixation of the prosthesis.

**Methods:**

A total of 144 patients (154 hips) who underwent total hip arthroplasty using the modular prosthesis were retrospectively investigated. For the cohort study, 97 patients (104 hips) were included. The femoral component survival rate and sleeve fixation were assessed at a minimum follow-up of 5 years. Patients were divided into 2 groups, including stable and unstable fixation groups, by sleeve fixation. Clinical and radiographic outcomes were compared.

**Results:**

The Kaplan-Meier survival rate at 9 years was 93% with revision for any reason as the endpoint in study cohort. The reasons for revision were recurrent dislocation (1 hip) and aseptic loosening of the stem (5 hips). A total of 88 hips (84.6%) showed stable fixation, and 16 hips (15.4%) showed unstable fixation at final follow-up. There was no significant difference in clinical outcomes between the 2 groups at final follow-up. The canal flare index was significantly higher, and the canal filling ratio was significantly lower in the unstable fixation group.

**Conclusion:**

Although the modified modular prosthesis was useful for treating anatomically difficult patients, we need to pay attention to both proximal/distal mismatch of the intramedullary canal and the canal filling ratio to achieve stable fixation and good long-term results.

## Introduction

Even though monoblock total hip arthroplasty (THA) has shown excellent and reliable long-term results [1, 2], it has been difficult to treat complicated cases, such as developmental dysplasia of the hip (DDH), post-osteotomy of the hip, and higher posterior pelvic tilt. In Japan, more than 80% of hip joint osteoarthritis patients had DDH [3, 4]. Selection of the femoral component is one of the most important points to gain primary and secondary fixation and prevent dislocation arising from lesser or greater femoral anteversion, a narrower medullary canal [5], bone deformity of the acetabulum and femur, and higher pelvic tilt. A modular prosthesis was one of the options to treat such cases, even though there were possible complications, such as fretting, corrosion, dissociation, and failure at the junction of the modular system [6–8].

In 1984, the S-ROM system (DePuy, Warsaw, IN) was developed as a modular prosthesis to treat these various types of anatomical deformities. This prosthesis is composed of a sleeve and stem body so that surgeons can change stem anteversion as they like to prevent dislocation. In addition, surgeons can treat various intramedullary canal shapes to select the stem and sleeve separately from various sizes. In 2004, the S-ROM-A prosthesis was developed as a modification of the S-ROM stem for Asian patients who have a smaller body and narrower medullary canal. The S-ROM-A prosthesis has a shorter stem length with a bullet tip to reduce thigh pain and periprosthetic fracture and a 9/10 neck taper to reduce implant impingement.

There are some reports of the short- to long-term results of the S-ROM [9–12] and S-ROM-A [13–16] prosthesis for primary THA. Although excellent fixation rates (99.5–100%) have been reported, with no evidence of aseptic loosening, we had cases of unstable fixation and revision due to aseptic loosening. The purpose of this study was to clarify the causes for unstable fixation of the stem. The hypothesis was that the canal filling ratio of the sleeve and stem is associated with unstable fixation.

## Material and methods

All procedures performed in this study involving human participants were in accordance with the ethical standards of the institutional and/or national research committee and with the 1964 Helsinki Declaration and its later amendments or comparable ethical standards.

The Institutional Review Board approved the study and waived informed consent.

**Fig. 1:**
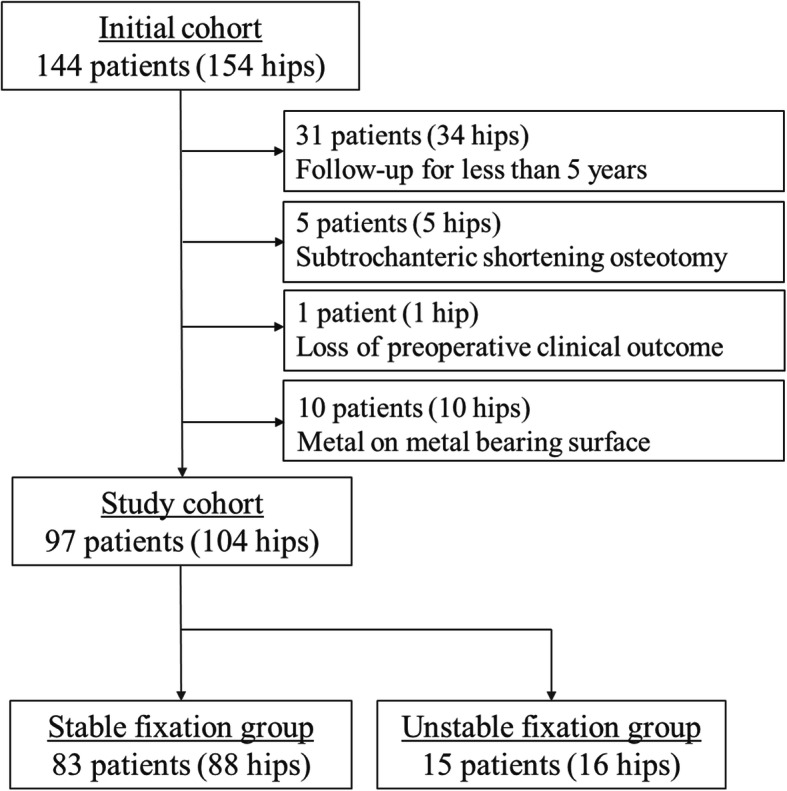
Flowchart of the study cohort

The medical records of 144 patients (154 hips) who underwent primary THA using the S-ROM-A prosthesis between July 2009 and January 2014 were retrospectively reviewed. Patients who were followed up less than 5 years (34 hips), those who underwent subtrochanteric shortening osteotomy (5 hips), and those with a metal on metal bearing surface (10 hips) were excluded. The pre-operative clinical outcome of one patient was not available. Therefore, 97 patients (male, *n* = 20; female, *n* = 77; mean age at surgery, 62 (36–84) years; follow-up, 8 (5–10) years; hips, *n* = 104) were assessed (Fig. 1). The underlying diagnoses were osteoarthritis (*n* = 7; primary, *n* = 89; secondary), rheumatoid arthritis (*n* = 1), trauma (*n* = 2), rapidly destructive coxarthropathy (*n* = 3), and osteonecrosis (*n* = 2).

The criteria for using the stems were severe deformity including DDH (Crowe 2 or 3) and post-osteotomy, femoral anteversion < 10° or more than 40°, pelvic tilt angle > 25° [17], or patients at high risk for dislocation, such as those with mental disorders or Parkinson’s disease. Of all the patients, 87 (83.4%) were diagnosed with osteoarthritis secondary to DDH, 21 patients (20.2%) had undergone acetabular osteotomy, and 14 patients (13.5%) had undergone femoral osteotomy. Overall, 36 hips (34.6%) had severe DDH (Crowe 2 or 3), 36 hips (34.6%) had lesser or greater femoral anteversion, and 14 hips (13.5%) had higher posterior pelvic tilt (Table 1).

**Table 1:** Patients’ demographics and clinical outcomes

	Total population (*n* = 104)		Stable group (*n* = 88)		Unstable group (*n* = 16)		*p* value
	mean ± SD	(range)	mean ± SD	(range)	mean ± SD	(range)	
Age (years)	62 ± 11	(36–84)	64 ± 10	(42–84)	55 ± 9	(36–68)	0.0015
Sex (M/F)	20/97		17/71		5/11		
Height (cm)	154 ± 9	(126–174)	154 ± 9	(126–174)	155 ± 8	(144–167)	0.77
Weight (kg)	55 ± 10	(33–79)	55 ± 9	(33–79)	60 ± 12	(38–78)	0.0869
BMI	23 ± 4	(17–34)	23 ± 3	(17–33)	25 ± 5	(18–34)	0.0837
Follow-up period (years)	7.5 ± 1.2	(5–9.7)	7.6 ± 1.2	(5–9.7)	7.3 ± 1.2	(5–8.9)	0.3
Diagnosis (hips)							
Primary osteoarthritis	7		5		2		
Osteoarthritis secondary to DDH	87		73		14		
Crowe classification (1,2,3,4)	52, 28, 7, 0		42, 24, 7, 0		10, 4, 0, 0		
Osteoarthritis secondary to trauma	2		2		0		
Rheumatoid arthritis	1		1		0		
Osteonecrosis	2		2		0		
Rapidly destructive coxarthropathy	3		3		0		
Trauma	2		2		0		
Acetabular osteotomy (hips)	21		16		5		
Femoral osteotomy (hips)	14		10		4		
Preoperative JOA score							
Total			50 ± 12	(22–77)	51 ± 6	(43–61)	0.31
Pain			16 ± 8	(0–30)	17 ± 7	(10–30)	0.65
ROM			12 ± 4	(2–20)	10 ± 5	(3–20)	0.1395
Walk			9 ± 4	(0–18)	10 ± 4	(5–15)	0.46
ADL			12 ± 3	(4–20)	14 ± 3	(10–20)	0.0495
JOA score at final follow-up							
Total			83 ± 11	(43–100)	82 ± 14	(55–100)	0.75
Pain			37 ± 5	(20–40)	34 ± 6	(20–40)	0.0946
ROM			17 ± 3	(4–20)	14 ± 5	(6–20)	0.0605
Walk			13 ± 5	(0–20)	16 ± 4	(5–20)	0.0739
ADL			16 ± 3	(4–20)	17 ± 3	(12–20)	0.44

*SD* standard deviation, *BMI* body mass index, *DDH* developmental dysplasia of the hip, *ROM* range of motion, *ADL* activities of daily living

*P* values for the comparisons between the stable and unstable groups

### Surgical data

Surgery was performed through the Hardinge approach in 83 hips (80%) and the posterior approach in 21 hips (20%) in the lateral decubitus position. The acetabular component was a Pinnacle-A (DePuy) and the STD-CP (cemented cup, JMM, Osaka, Japan) in 96 and eight hips, respectively. The mean outer diameter of the cups was 51 (44–58) mm. All bearing surfaces were cobalt-chromium on highly cross-linked polyethylene. The head diameters were 28, 32, and 36 mm in 46, 57, and one hips, respectively. Before surgery, pre-operative planning was performed using 2D templating on the anteroposterior view. The size of the distal stem was determined when distal reaming contacted cortical bone, and the size of the sleeve was determined after proximal and calcar reaming during surgery. If the leg length would be too long, the smaller size of sleeve was used.

### Implant survival rate

The implant survival rate of 97 patients was calculated by the Kaplan-Meier method with revision for any reason as the endpoint.

### Sleeve fixation

**Fig. 2:**
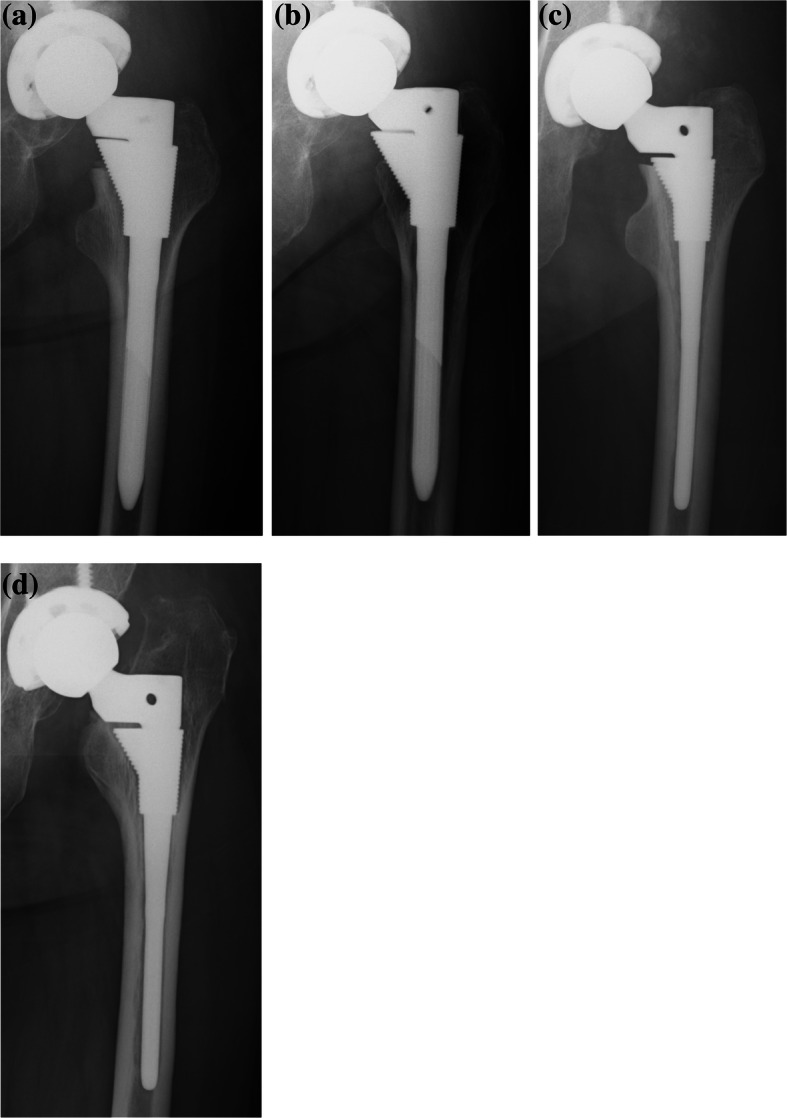
Stem with stable fixation and unstable fixation 1 week (**a**, **c**) and 5 years (**b**, **d**) after surgery. Spot welds around the distal sleeve were confirmed in the stable fixation (**b**). Extensive radiolucent line around the sleeve was confirmed with progressive subsidence and varus stem alignment change in the unstable fixation (**d**)

Sleeve fixation was classified as stable fixation or unstable fixation following Engh’s classification [18, 19]. Stable fixation was defined as no or slight radiolucent lines around the sleeve. Spot welds around the distal sleeve were considered evidence of bone ingrowth. Unstable fixation was defined as an extensive radiolucent line around the sleeve (> 50%) with progressive subsidence or migration (Fig. 2).

### Groups

Patients were separated into two groups including 88 with stable fixation and 16 with unstable fixation of the stem at final follow-up. We compared clinical outcomes, stem alignment, and the canal filling ratio (Fig. 1).

### Clinical evaluation

The Japanese Orthopaedic Association (JOA) hip score was evaluated pre-operatively, post-operatively at 6 months, and then annually. The JOA score ranges from 0 to 100 points and is used to evaluate clinical outcomes of the hip joint. It is composed of four parameters: pain (0 to 40 points), range of motion (0 to 20 points), walking ability (0 to 20 points), and daily living activities (0 to 20 points). The JOA hip score of revision cases was determined at final follow-up before revision.

### Radiographic evaluation

**Fig. 3:**
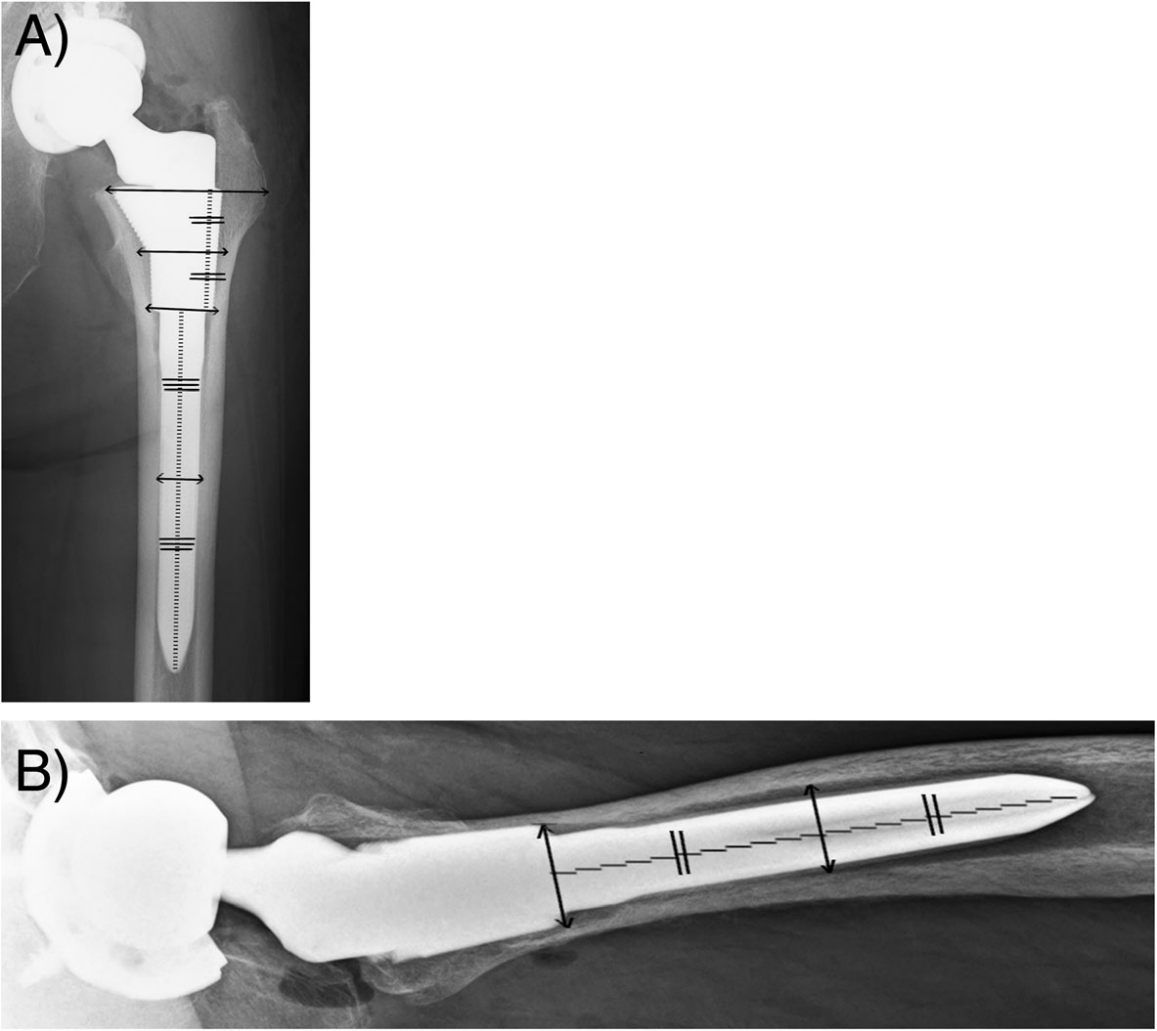
Measurement levels of the canal filling ratio on the anteroposterior (**a**) and lateral views (**b**). The canal filling ratio was defined as the width of the stem divided by the width of the intramedullary canal, measured at the each level

All radiological data were measured three times by a single author (KK), and the average was taken. Interobserver agreement of canal filling ratio was measured by KK, , and TM. Regarding the canal flare index (CFI) and the canal filling ratio measurement, cases of post-osteotomy of the femur (12 stable group and 2 unstable group) were excluded because their intramedullary canals were unclear. The CFI was defined as the width of the intramedullary canal at 20 mm above the lesser trochanter divided by the width of the isthmus [20]. Canal shape was separated into champagne flute (CFI > 4.7), normal (CFI 3.0–4.7), and stovepipe (CFI < 3.0). Coronal stem alignment was evaluated at post-operative 1 week and at final follow-up; > 2° was defined as varus, and valgus was defined as against the bone axis. Coronal stem alignment change was calculated as stem alignment at final follow-up subtracted by stem alignment at post-operative one week. Stress shielding in the stable fixation group was evaluated using Engh’s classification [18]. The presence of a radiolucent line around the sleeve was assessed on the anteroposterior view of the X-ray at post-operative 1 year and at final follow-up. The canal filling ratio was defined as the width of the stem divided by the width of the intramedullary canal [21], measured at the level of the bone resection line, at half the height of the sleeve, at the distal end of the sleeve, at half the height of the distal stem on the anteroposterior view and the distal end of the sleeve, and at half the height of the distal stem on the lateral view taken within 1 week after surgery (Fig. 3). Periprosthetic osteolysis was defined as a rounded or scalloped lesion around the implant at least 1 mm wide, and it increased in size [22].

### Statistical analysis

Intergroup differences in dichotomous continuous or binary variables were tested for significance using the Wilcoxon test or Fisher’s exact test. *P* < 0.05 was considered significant. Interobserver agreement was calculated using intraclass correlation coefficients (ICCs) and interpreted as follows [23]: < 0.4 poor; 0.4–0.59 fair; 0.6–0.74 good; and 0.75–1.00 excellent.

## Results

The 5-year and 9-year implant survival rates were 99% and 93%, respectively, using the endpoint of revision for any reason. The stem revision rate was 5.8% (6 hips), and the mean duration to revision was 5.8 (4.4–7.4) years. The reasons for revision were recurrent dislocation (1 hip) and aseptic loosening of the stem (5 hips). Stable sleeve fixation was seen in 88 hips (84.6%), with unstable fixation in 16 hips (15.4%). Intraoperative fracture occurred in eight hips (7.7%). Recurrent dislocation occurred in one hip (1%), and post-operative periprosthetic fracture occurred in one hip (1%). There were no infections and no evidence of osteolysis.

In the stable fixation group, age was significantly younger (*P* = 0.0015). Although the pre-operative JOA scores for activities of daily living (*P* = 0.0495) were significantly higher, there was no significant difference in JOA scores at final follow-up (Table 1). The mean CFI was significantly higher in the unstable group (*P* = 0.0262). The intra- and interobserver agreements were good to excellent for canal filling ratio measurements (Table 2). The canal filling ratio at each level on the anteroposterior and lateral view was significantly lower in the unstable group (Table 3).

**Table 2:** Intra- and inter-observer agreements for the canal filling ratio

	Intra-observer		Inter-observer	
	ICC	95% CI	ICC	95% CI
Resection line	0.98	0.96–0.99	0.82	0.70–0.90
Half the height of the sleeve	0.98	0.96–0.99	0.77	0.63–0.88
Distal end of the sleeve	0.86	0.75–0.93	0.81	0.68–0.90
Half the height of the stem	0.97	0.95–0.99	0.78	0.61–0.88
Distal end of the sleeve on the lateral view	0.97	0.95–0.99	0.71	0.53–0.84
Half the height of the stem on the lateral view	0.94	0.90–0.97	0.81	0.68–0.90

*ICC* intra-class correlation coefficient, *CI* confidence interval

**Table 3:** Radiographic evaluation

	Stable group (*n* = 76)		Unstable group (*n* = 14)		*P* value
	mean ± SD	(range)	mean ± SD	(range)	
Canal flare index	3.6 ± 0.7	(2.2–5.5)	4.3 ± 1.0	(2.7–6.1)	0.0262
Canal filling ratio (%)					
Resection line	65.7 ± 7.7	(51.1–86.7)	60.6 ± 5.7	(51.1–71.8)	0.0228
Half the height of the sleeve	69.3 ± 9.3	(44.8–90.0)	61.3 ± 9.3	(50.9–87.1)	0.002
Distal end of the sleeve	82.5 ± 8.1	(61.8–95.5)	71.0 ± 4.8	(66.7–85.6)	<0.0001
Half the height of the stem	86.9 ± 5.3	(73.8–95.9)	80.1 ± 6.1	(70.4–89.8)	0.0005
Distal end of the sleeve on the lateral view	80.2 ± 8.5	(52.7–95.8)	68.8 ± 8.8	(47.7–84.8)	<0.0001
Half the height of the stem on the lateral view	69.4 ± 6.4	(54.5–85.9)	63.5 ± 9.5	(39.3–77.6)	0.0197
	Hips		Hips		
Champagne flute/normal/stovepipe	6/59/11		7/5/2		
	Stable group (n=88)		Unstable group (*n* = 16)		*P* value
	Hips	(%)	Hips	(%)	
Preoperative stem alignment					
Neutral/Varus/Valgus	87/1/0		16/0/0		
Stem alignment at final follow up					
Neutral/varus/valgus	85/3/0		12/4/0		
Stem alignment change					
< 2°	87	98.9	9	56.2	
≥ 2° varus	1	1.1	7	43.8	<0.0001
Radiolucent line around the sleeve (> 50%)					
Postoperative 1 year	1	0.9	18	100	<0.0001
Final follow-up	0	0	18	100	<0.0001
Stress shielding (grade 3/grade 4)	5/4	4.5/3.6			

*SD* standard deviation

**Fig. 4:**
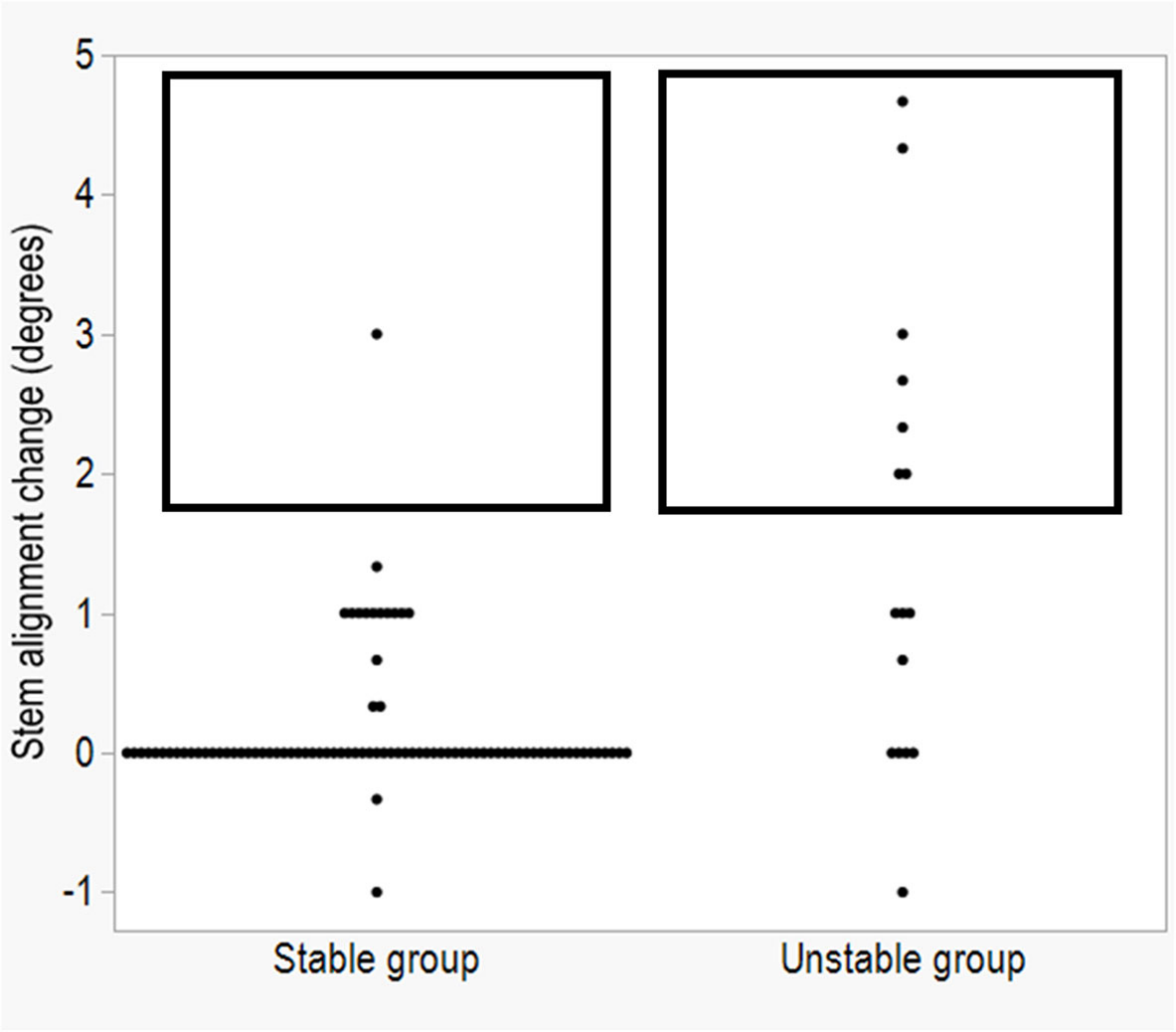
Stem alignment change in the stable and unstable groups. Rectangle, coronal stem alignment changed ≥ 2° varus

One hip (1%) in the stable group and none in the unstable group had varus coronal stem alignment at post-operative one week. At final follow-up, three hips (3.4%) in the stable group and four hips (25%) in the unstable group had varus coronal stem alignment (Table 3). Stem alignment changed ≥ 2° in one hip (1%) in the stable group and seven hips (43.8%) in the unstable group (Fig. 4, Table 3). Grades 3 and 4 severe stress shielding was seen in five hips (5.7%) and four hips (4.6%), respectively, in the stable group at final follow-up.

## Discussion

### Femoral component survival rate and revision

To the best of our knowledge, this is the first report of unstable fixation and aseptic loosening using the modified S-ROM prosthesis in primary THA. So far, there have been some reports of the mid- to long-term results of the S-ROM and S-ROM-A prostheses in primary THA. Le et al. and Biant et al. reported that no femoral components were revised with mean follow-up durations of 17 years and 10 years, respectively, using the S-ROM prosthesis [11, 12]. Sato et al. reported a 10-year femoral component survival rate of 84% for the S-ROM-A prosthesis, and the reason for all revisions was adverse reaction to the metal debris (ARMD) [16]. They used metal on metal, metal on polyethylene, and ceramic on polyethylene bearing surfaces, and the revision rates were 10.3%, 2.5%, and 0% respectively. In the present study, the 9-year femoral component survival rate was 93%. The reasons for femoral component revision were recurrent dislocation (1 hip) and aseptic loosening (5 hips). In the former, the femoral component was revised using the S-ROM-A prosthesis to change anteversion, and no dislocation occurred after revision. In the latter, the femoral components were revised using cemented stems.

### Stem fixation

Cameron reported that they used the S-ROM prosthesis in 202 primary THA cases, and there was no evidence of loosening with a follow-up period of 2 to 8 years [9]. Le et al. reported that all 31 hips achieved bone ingrowth with a minimal follow-up of 15 years using the S-ROM prosthesis [11]. Kido et al., Tamegai et al., and Hozumi et al. reported that bone ingrowth of the sleeve was achieved in 97.1% with a mean follow-up of 2.3 years [13], 99.5% with a mean follow-up of 3.3 years [14], and 97% with a mean follow-up of 4.6 years, respectively [15]. Sato et al. reported excellent mid-term fixation of the S-ROM-A prosthesis. All stems showed bone ingrowth in 331 hips [16]. Compared to these previous reports, the present results for stem fixation were worse. Sixteen hips (15.4%) showed extensive radiolucent lines around the sleeve (> 50%) without spot welds at final follow-up. The S-ROM-A prosthesis was designed to be modular and to achieve fit and fill at both the metaphysis and the diaphysis [9]. The proximal parts including the sleeve and stem needed optimal fit and fill to achieve primary fixation of the stem against vertical force. The distal diameter of the stem was also important because it affected proximal stem diameter and generated torsional stability [24]. In the present study, the CFI was significantly higher and the canal filling ratio was significantly lower in the unstable group, and all stems showed extensive radiolucent lines around the sleeve from post-operative 1 year. These findings showed that we failed to achieve primary fixation of the femoral component due to proximal/distal mismatch of the intramedullary canal of the proximal femur using small sleeves at the metaphysis and using an undersized stem following insufficient reaming at the diaphysis. We should have reamed the distal canal to achieve sufficient filling and used sleeves with as large a diameter as possible and pushed them in tightly.

### Pain and stem alignment change

Aghayev et al. reported that painful hip increased from 15 to 80% 2 years before revision [25]. In the present study, the post-operative JOA score of pain was not significantly lower in the unstable group, but all revision cases for aseptic loosening had pain during walking. Two of six revision hips (33%) had continuous pain after surgery, and three hips (50%) had pain for more than two years before revision.

Regarding the relationship between pain and stem alignment change, three of seven hips (63%) whose stem alignment gradually changed ≥ 2° varus in the unstable group had pain, but the others (37%) had no pain or only discomfort. Of the eight hips, six were in female patients, and the others (same patient) had protrusion of the acetabulum and a stovepipe femur.

There was a risk of periprosthetic fracture or perforation due to varus stem alignment change at the lateral cortex of the femur. These findings showed that pain was of course important, and sometimes the presence of a radiographic sign was the only useful monitor; it was necessary to revise the stem of female cases and poor bone quality cases with unstable fixation of the sleeve to prevent periprosthetic fracture.

### Limitations

The present study has several limitations. First, this retrospective study did not allow a patient-based assessment of pain and function. Second, in cases of post-osteotomy of the femur, the canal flare index and canal filling ratio (12 stable group and 2 unstable group) were not measured because their intramedullary canals were unclear.

## Conclusion

The S-ROM-A prosthesis was useful to treat anatomically difficult patients as long as surgeons kept its concept in mind. Both the distal stem and the proximal sleeve need to be as large as possible to achieve stable fixation and good long-term results with this prosthesis.

## Data Availability

The datasets during and/or analyzed during the current study are available from the corresponding author on reasonable request.

## Abbreviations

THA: TOTAL hip arthroplasty
DDH: Developmental dysplasia of the hip
JOA: The Japanese Orthopaedic Association
CFI: Canal flare index
ICCs: Intraclass correlation coefficients
